# Dehydration reduces stroke volume and cardiac output during exercise because of impaired cardiac filling and venous return, not left ventricular function

**DOI:** 10.14814/phy2.14433

**Published:** 2020-06-15

**Authors:** Kazuhito Watanabe, Eric J. Stöhr, Koichi Akiyama, Sumie Watanabe, José González‐Alonso

**Affiliations:** ^1^ Centre for Human Performance, Exercise and Rehabilitation Brunel University London Uxbridge UK; ^2^ Faculty of Education and Human Studies Akita University Akita Japan; ^3^ Cardiff School of Sport and Health Sciences Cardiff Metropolitan University Cardiff UK; ^4^ Department of Medicine Division of Cardiology Columbia University Irving Medical Center New York City NY USA; ^5^ Department of Anesthesiology Yodogawa Christian Hospital Osaka Japan; ^6^ Division of Sport, Health and Exercise Sciences Department of Life Sciences Brunel University London Uxbridge UK

**Keywords:** blood flow, intraventricular pressure gradients, left ventricular volumes, twist and untwisting rate

## Abstract

Dehydration accrued during intense prolonged whole‐body exercise in the heat compromises peripheral blood flow and cardiac output ($\dot{Q}$). A markedly reduced stroke volume (SV) is a key feature of the dehydration‐induced cardiovascular strain, but whether the lower output of the heart is mediated by peripheral or cardiac factors remains unknown. Therefore, we repeatedly quantified left ventricular (LV) volumes, LV mechanics (LV twist, a marker of systolic muscle function, and LV untwisting rate, an independent marker of LV muscle relaxation), left intra‐ventricular pressure gradients, blood volume and peripheral blood flow during 2 hr of cycling in the heat with and without dehydration (DEH: 4.0 ± 0.2% body mass loss and EUH: euhydration control, respectively) in eight participants (three females and five males). While brachial and carotid blood flow, blood volume, SV, LV end‐diastolic volume (LVEDV), cardiac filling time, systemic vascular conductance and $\dot{Q}$ were reduced in DEH compared to EUH after 2 hr, LV twist and untwisting rate tended to be higher (*p* = .09 and .06, respectively) and intra‐ventricular pressure gradients were not different between the two conditions (*p* = .22). Furthermore, LVEDV in DEH correlated strongly with blood volume (*r* = .995, *p* < .01), head and forearms beat volume (*r* = .98, *p* < .05), and diastolic LV filling time (*r* = .98, *p* < .05). These findings suggest that the decline in SV underpinning the blunted $\dot{Q}$ with exercise‐induced dehydration is caused by compromised LV filling and venous return, but not intrinsic systolic or diastolic LV function.

## Graphical abstract

Dehydration reduces stroke volume and cardiac output during intense prolonged exercise in the heat, but the underlying peripheral and cardiac mechanisms are not fully understood. We show that the reduced stroke volume accounting for the impaired cardiac output is closely associated with diminished left ventricular filling secondary to decreased peripheral blood flow and volume, compromised venous return, and reduced cardiac filling time. These findings highlight the importance of peripheral mechanisms in cardiac performance during exercise.

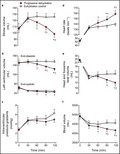

## 1 INTRODUCTION

Progressive dehydration accrued during intense prolonged whole‐body exercise in the heat reduces stroke volume (SV), cardiac output ($\dot{Q}$), arterial blood pressure and blood flow to the active skeletal muscle, skin, and brain and concomitantly increases core temperature, heart rate (HR) and total peripheral resistance (TPR) (Trangmar and González‐Alonso, 2017; Trangmar and González‐Alonso, 2019). The progressive fall in $\dot{Q}$ owing to SV reduction is a key feature of the global cardiovascular strain induced by dehydration and hyperthermia (González‐Alonso, 1998; González‐Alonso et al., 1995, 1998; Hamilton et al., 1991; Montain and Coyle, 1992b; Montain et al., 1998; Sawka et al., 1979). However, the peripheral and cardiac mechanisms underlying the blunted $\dot{Q}$ with progressive dehydration and hyperthermia during prolonged exercise are not fully understood.

One plausible explanation is that the decline in $\dot{Q}$ with the development of dehydration and hyperthermia is due to the reduction in left ventricular (LV) filling induced by a loss in blood volume, largely dehydration‐induced plasma volume loss. However, previous studies have shown that substantial blood volume losses decrease SV but do not reduce $\dot{Q}$ when exercise is performed in a cold environment (González‐Alonso et al., 2000) and when the metabolic demand is low such as at rest and during exercise recruiting small muscle mass due to a concomitant increase in HR (Pearson et al., 2013; Stöhr et al., 2011a). Moreover, Montain and Coyle (1992a) showed that plasma volume expansion in dehydrated and hyperthermic individuals restores one‐half of the decline in SV and attenuates the increase in HR such that $\dot{Q}$ is maintained. It therefore seems that a reduction in blood volume alone only explains part of the SV decline and that the compromised $\dot{Q}$ is likely due to the interaction of several cardiac and/or peripheral factors.

Reduced venous return secondary to enhanced peripheral vasoconstriction and reduced tissue blood flow might be another important peripheral factor involved in the compromised SV and $\dot{Q}$. In support of this mechanism, it has been demonstrated that limb vasoconstriction via intra‐arterial infusion of adenosine and the sympathomimetic agent tyramine (Rosenmeier et al., 2008) and the combined blockade of prostaglandins and nitric oxide using N^G^‐monomethyl‐L‐arginine and indomethacin infusion (Mortensen et al., 2007) lead to proportional reductions in limb blood flow and $\dot{Q}$. Based on these findings, it is conceivable that the gradual fall in $\dot{Q}$ with dehydration and hyperthermia during prolonged exercise may accompany not only lowered blood volume but also peripheral vasoconstriction‐mediated restrictions in peripheral perfusion, venous return, and thus LV filling. To the best of our knowledge, however, the impact of dehydration and hyperthermia on LV volume along with systemic and peripheral hemodynamics has never been investigated during intense prolonged exercise.

Another possible scenario to explain the compromised $\dot{Q}$ in the dehydrated and hyperthermic state might be an impaired LV systolic or diastolic function. Previous studies have shown that LV diastolic function is depressed immediately after long‐duration exercise with mild‐to‐moderate (~0.5%–2%) body mass loss (George et al., 2005; Nottin et al., 2012; Oxborough et al., 2010) and that both LV systolic and diastolic functions are blunted following ultra‐endurance exercise accompanied by substantial (~3%–4.5%) body mass loss (Douglas et al., 1987; Niemelä et al., 1984; Nottin et al., 2009; Whyte et al., 2000). However, it is unclear whether those findings in the post‐exercise period apply to what happens during exercise given the large differences in cardiovascular loads between conditions (Wilhelm et al., 2016). Our earlier studies during single leg knee‐extensor exercise with stable or increased peripheral blood flow and $\dot{Q}$ showed that systolic and diastolic LV twist mechanics are maintained or even mildly enhanced with significant dehydration (3.5% body mass loss), but low physiological load (Pearson et al., 2013; Stöhr et al., 2011a). It remains unknown, however, whether LV systolic and diastolic function are preserved or impaired during intense prolonged whole‐body exercise in the heat causing much greater physiological load and dehydration‐induced cardiovascular strain. Systolic and diastolic LV mechanics and intraventricular pressure gradients reflect the mechanical events of the cardiac cycle (Beyar and Sideman, 1984; Courtois et al., 1988; Notomi et al., 2005) and thus can provide insight into the intrinsic cardiac factors influencing SV with changes in hydration status during prolonged exercise.

Accordingly, the aim of this study was to investigate the impact of hydration status and environmental heat stress on systemic and peripheral hemodynamics, LV volume, LV mechanics and left intra‐ventricular pressure gradients during prolonged whole‐body exercise inducing significant cardiovascular strain compared to exercise with physiological stability, and to provide insights into the central and peripheral mechanisms underpinning dehydration‐induced decline in $\dot{Q}$. To this aim, we manipulated hydration status to test the hypothesis that combined dehydration and hyperthermia during prolonged cycling in the heat would compromise SV and $\dot{Q}$ in close association with restrictions in LV end‐diastolic volume (LVEDV) and peripheral perfusion rather than intrinsic cardiac factors such as systolic and diastolic LV mechanics and intraventricular pressure gradients.

## 2 METHODS

### 2.1 Subjects

Eight trained cyclists and triathletes (3 females and 5 males) with a mean (±*SD*) age of 31 ± 6 years, height of 176 ± 6 cm, body mass of 70 ± 10 kg and $\dot{V}$O_2peak_ during semi‐recumbent cycling of 3.8 ± 0.6 L/min, participated in the study. They arrived at the laboratory postprandial with a normal hydration status and were required to abstain from strenuous exercise and alcohol intake for 24 hr and caffeine consumption for 12 hr. The study was approved by the Brunel University London Research Ethics Committee (5449‐TISS‐Feb/2017‐ 6276‐2 and 5449‐A‐Mar/2017‐ 6957‐1) and was carried out in accordance with the Declaration of Helsinki. All participants provided written informed consent prior to commencement of the study.

### 2.2 Experimental design

Participants visited the laboratory on four occasions, consisting of two familiarization sessions and two experimental trials. On the first familiarization visit, an incremental exercise test on a semi‐recumbent cycle ergometer (Lode Angio) was conducted to determine the maximal work rate (*W*_max_) and $\dot{V}$O_2peak_ during semi‐recumbent exercise. After 15–20 min break, participants were familiarized to the experimental protocol by cycling in the semi‐recumbent position for 1 hr at 50%–55% *W*_max_ with the pedal cadence maintained between 70–90 rpm in an environmental chamber set at 35°C and 50% relative humidity with convective fan cooling provided. On the second familiarization visit, they cycled for 2 hr in the same exercise and environmental conditions. During this session, echocardiography and ultrasound hemodynamic measurements (see below for further details) were performed to familiarize the participants with the primary measures of the main experimental trials.

**Figure 1:**
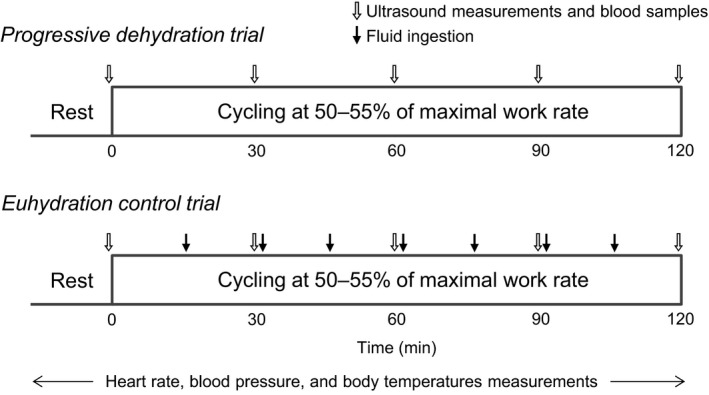
Schematic representation of the experimental protocols. Participants completed the progressive dehydration and euhydration control trials at the same exercise intensity on two different days, separated by a week

**Figure 2:**
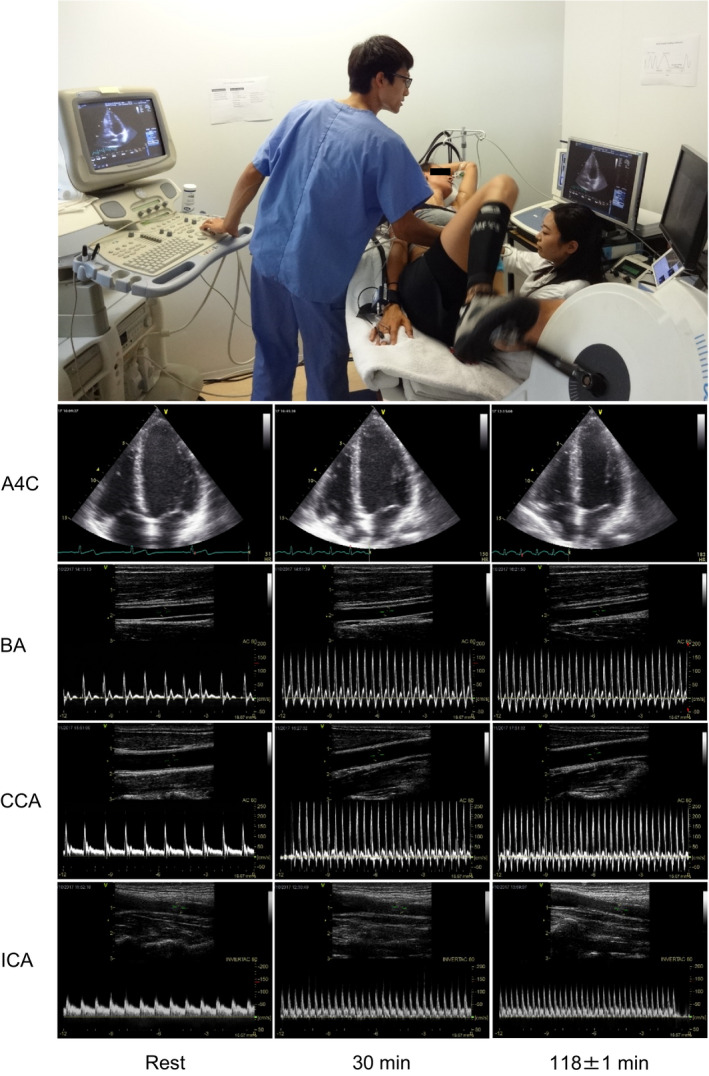
Examples of echocardiographic and Doppler ultrasonographic assessments during prolonged submaximal semi‐recumbent cycling. The photo depicts one of the participants in the study. Representative images of apical 4‐chamber view at end diastole (A4C) and vessel and blood velocity recordings at the brachial artery (BA), common carotid artery (CCA), and internal carotid artery (ICA), at rest and during exercise (30 and 118 ± 1 min) in the progressive dehydration trial are shown

In the two experimental trials (visits 3 and 4), participants performed prolonged (~2 hr) of semi‐recumbent cycling at 50%–55% of *W*_max_ (141 ± 7 W) in the same environmental conditions as in the familiarization sessions with (euhydration control) and without (progressive dehydration) fluid replacement. Trials were separated by a week and were randomly assigned and counterbalanced across participants. In the progressive dehydration trial, participants did not consume fluid during the prolonged exercise. In this trial, two subjects reached volitional exhaustion early (110 and 113 min, respectively), however, final measurements were obtained. In the euhydration trial, participants exercised for the same period of time (118 ± 1 min) but hydration was maintained through fluid ingestions according to the participants’ body mass loss (1.4 ± 0.1 L/hr). Fluid was provided in seven equal aliquots every 15 min during exercise in the form of 0.3% NaCl solution. In both experimental trials, LV volumes and functions and arm and head hemodynamics were assessed using an ultrasound system and blood samples were taken at rest and every 30 min during exercise. HR, blood pressure, and body temperatures were recorded continuously. Body mass was measured before and immediately after exercise each trial. Full depiction of the experimental protocol and physiological measurements is presented in Figures 1 and 2.

### 2.3 Cardiac function

#### 2.3.1 LV volume and function

LV volume and function were assessed using an ultrasound system (Vivid 7 Dimension; GE Healthcare) equipped with a sector array probe (M4S) according to current guidelines (Lang et al., 2015). At rest and every 30 min during the exercise, echocardiographic images of five consecutive cardiac cycles were obtained during 45° left lateral tilt of the semi‐recumbent cycle ergometer and saved at natural end‐expiration to maximise the image quality. All cardiac data are presented as the average of three consecutive cardiac cycles. Participants were familiarized with the exercise in that position and brief periods of end‐expiratory breath‐hold during exercise in the first 2‐familiarization visits. Care was taken to ensure that all ultrasound settings, including image depth and frame rates, were kept constant within subjects. Apical four‐chamber and two‐chamber images were recorded and LVEDV, end‐systolic volumes (LVESV) and SV were analyzed using the Simpson's biplane method using the manufacturer's software (EchoPAC PC version 112; GE Healthcare). $\dot{Q}$ was calculated as the product of HR and SV. TPR was calculated as mean arterial pressure (MAP)/$\dot{Q}$. LV filling time was assessed using pulsed wave tissue Doppler imaging of the septal mitral annular velocity (Alam et al., 1999; Stöhr et al., 2011b).

#### 2.3.2 Systolic and diastolic LV mechanics

Systolic and diastolic LV mechanics were assessed from 2D parasternal short‐axis images recorded at the mitral valve and apical level as described in detail previously (Stöhr et al., 2016). In brief, images at the mitral valve level (basal level) were standardized by ensuring that the images were taken as cranially as possible and the full‐thickness of the myocardium was imaged throughout the cardiac cycle. Apical short‐axis views were obtained by identifying the standardized apical four‐chamber location, and then tilting the probe into the short‐axis plane and moving the probe as little cranial as possible, whilst ensuring a circular view without luminal obliteration at end‐systole. A single focal point was positioned in the center of the ventricular cavity for all short‐axis images. Images were analyzed off‐line for two‐dimensional speckle tracking‐derived LV twist mechanics. From the raw speckle tracking output, data were interpolated to 600 points at equidistant time intervals in systole and diastole, respectively, as previously recommended (Burns et al., 2008). The frame‐by‐frame basal rotation (degrees) and rotation velocity (degrees/s) data were subtracted from apical rotation and rotation velocity data respectively to determine peak systolic LV twist (degrees) and early diastolic untwisting rate (degrees/s). Similarly, systolic radial and circumferential strain (both %) were quantified from speckle tracking analysis of parasternal short‐axis images, and longitudinal strain (%) was obtained from the analysis of apical four‐chamber images.

#### 2.3.3 Intra‐ventricular pressure gradients

We quantified left intraventricular pressure gradients from the LV base to the apex in early diastole using the validated approach based on color‐Doppler M‐mode images in the apical four‐chamber view (Greenberg et al., 2001; Notomi et al., 2006; Rovner et al., 2003). The Doppler M‐mode cursor was aligned with the diastolic inflow streamline and the images were analyzed with an image processing algorithm based on the one‐dimensional Euler equation. Peak left intraventricular pressure gradients were defined as the maximal pressure difference between the LV base (mitral annulus) and the LV apex.

### 2.4 Non‐exercising limb and head hemodynamics

Blood flow was measured at rest and every 30 min of exercise in the brachial artery (BA), common carotid artery (CCA), and internal carotid artery (ICA) as previously described (Kalsi et al., 2017; Trangmar et al., 2014) using a 10 MHz linear probe (10L, GE Healthcare). For the measurement, the participants’ right arm was extended and positioned on a table at the side of the cycle ergometer, and blood flow was obtained ~5 cm proximal to the antecubital fossa. The right CCA and ICA blood flows were measured ~1.5 cm proximal to and ~1.0–1.5 cm distal to the carotid bifurcation, respectively. Longitudinal images of the arteries were recorded when the intima‐media boundary was clearly visible, and artery diameters related to systole and diastole were measured as the largest and smallest diameters within each cardiac cycle, and then the mean diameter was calculated as systolic diameter × 1/3 + diastolic diameter × 2/3. BA, CCA, and ICA cross‐sectional areas (CSAs; in cm^2^) were estimated as follows: CSA = π × (mean diameter/2)^2^. Blood velocity was measured simultaneously with artery images using continuous pulsed‐wave Doppler at a frequency of 4.4 MHz, with an insonation angle consistently below 60 degrees and the sample volume extended to cover the entire vessel lumen. Continuous 12‐s blood velocity profiles were recorded and analyzed offline to calculate time‐averaged mean blood velocity (*V*_mean_) with the manufacturer's software (EchoPAC PC version 112; GE Healthcare). BA, CCA, and ICA blood flows were calculated as the product of the *V*_mean_ (in cm/s) and CSA (in cm^2^) and were multiplied by 60 to obtain values expressed in ml/min. BA, CCA, and ICA vascular conductances were calculated as BA blood flow/MAP, CCA blood flow/MAP, and ICA blood flow/MAP, respectively. Assuming that the amount of arterial inflow and venous outflow are the same during steady‐state condition, the amount of blood passing through the peripheral veins during each cardiac cycle (beat volume; in ml) was estimated by dividing blood flow in the CCA and BA by HR and was doubled and the added together to obtain an index of the head and forearms beat volume (Elstad et al., 2009; Trangmar and González‐Alonso, 2017).

### 2.5 Hematological parameters

Blood samples were taken via a venous cannula inserted into a superficial antecubital vein for subsequent measurements of hemoglobin (Hb) concentration via the azidemethemoglobin method (HemoCue^®^ Hb 201 + System, HemoCue AB) and hematocrit (Hct), measured in quadruplicates using standard sodium‐heparinized capillary tubes (micro‐haematocrit tubes, Hawksley) and centrifugation (5 min; HaematoSpin 1400, Hawksley) procedures. The percent changes in blood, red cell, and plasma volumes were calculated from the Hb and Hct values as described by Dill and Costill (1974). The absolute changes in blood, red cell, and plasma volumes (L) were then estimated using equations of Sawka et al. (1992). The placement of a venous catheter was not successful for one participant and therefore blood samples were obtained in seven participants.

### 2.6 Heart rate, arterial pressure, and body temperatures

HR was monitored via a three‐lead electrocardiogram. Arterial blood pressure was measured non‐invasively using finger photoplethysmography (Finometer, Finapres Medical Systems). The monitoring cuff was placed around the middle finger of the right hand, with the forearm and hand supported so that the cuff was at the vertical level of the heart. Core temperature (*T*_c_) was assessed using a commercially available rectal probe (RET‐1, Physitemp Instruments) inserted 15 cm past the sphincter muscle and connected to a thermocouple meter (TC‐2000, Sable Systems). Mean skin temperature (*T*_sk_) from four sites (standard weightings of chest, arm, thigh, and calf, (Ramanathan, 1964)) was obtained using a wireless monitoring system (iButton^®^, Maxim Integrated). Analog signals of the electrocardiogram, blood pressure waveform, and *T*_c_ were sampled at 1,000 Hz using a data acquisition unit (Powerlab 16/30, ADInstruments) and analyzed using an off‐line data analysis software (LabChart 8, ADInstruments).

### 2.7 Effective arterial elastance and LV end‐systolic elastance

It is known that the effective arterial elastance reflects the net arterial load imposed on the LV, whereas the LV end‐systolic elastance is considered to be an integrated measure of LV performance (Sagawa et al., 1977; Sunagawa et al., 1983). To obtain additional information on the cardiac afterload and myocardial contractility, we calculated noninvasive indexes of effective arterial elastance (0.9 × systolic blood pressure/SV) and LV end‐systolic elastance (0.9 × systolic blood pressure/LVESV), respectively (Chantler and Lakatta, 2012; Chantler et al., 2008).

### 2.8 Statistical analysis

Data are presented as means ± *SEM* unless otherwise stated. Differences in measured variables were assessed using a two‐way repeated‐measures analysis of variance (ANOVA) in which trial (progressive dehydration and euhydration control) and time (Rest, 30, 60, 90, and 118 ± 1 min) were the main factors. Tukey's honestly significant difference (HSD) was used as a *post hoc* test. The relationships between selected physiological variables were evaluated using Pearson's product‐moment correlation analysis. Statistical analyses were performed using IBM SPSS Statistics (version 24, IBM). *p*‐values < .05 were considered significant.

## 3 RESULTS

### 3.1 Hydration status and body temperature

Prolonged exercise without fluid ingestion resulted in a 4.0 ± 0.2% body mass reduction, reflecting a 2.0 ± 0.2 kg lower body mass at the end of exercise in the dehydration compared with the euhydration trial (*p* < .01, Table 1). The decline in body mass with dehydration was accompanied by reductions in blood volume (−388 ± 45 ml vs. euhydration, *p* < .01; Figure 4f) associated with decreases in both estimated red cell and plasma volumes (*p* < .01; Table 1). Concomitantly, *T*_c_ increased progressively in both trials but was on average 0.6 ± 0.1°C higher at the end of exercise in the dehydration compared with the euhydration trial (38.9 ± 0.2 vs. 38.3 ± 0.2°C, *p* < .01; Table 1). In contrast, *T*_sk_ remained stable throughout exercise and was similar in both trials (*p* > .05; Table 1).

**TABLE 1:** Hydration status and body temperature at rest and during prolonged exercise in the heat

	Exercise time (min)				
	Rest	30	60	90	118 ± 1
Body mass, kg					
Progressive dehydration	70.4 ± 3.5	—	—	—	67.6 ± 3.5^†^
Euhydration control	70.1 ± 3.6	—	—	—	69.7 ± 3.5
Body mass change, %					
Progressive dehydration	0	—	—	—	−4.0 ± 0.2^†^
Euhydration control	0	—	—	—	−0.6 ± 0.1
Red cell volume, L					
Progressive dehydration	1.85 ± 0.1	1.79 ± 0.1	1.79 ± 0.1	1.75 ± 0.1^†^	1.74 ± 0.1^†^
Euhydration control	1.85 ± 0.1	1.83 ± 0.1	1.80 ± 0.1	1.83 ± 0.1	1.82 ± 0.1
Plasma volume, L					
Progressive dehydration	3.12 ± 0.1	2.64 ± 0.1	2.58 ± 0.1^†^	2.48 ± 0.1*, ^†^	2.42 ± 0.1*, ^†^
Euhydration control	3.11 ± 0.1	2.74 ± 0.1	2.71 ± 0.1	2.75 ± 0.1	2.75 ± 0.1
Rectal temperature, °C					
Progressive dehydration	36.9 ± 0.1	37.8 ± 0.1	38.2 ± 0.05*	38.6 ± 0.1*, ^†^	38.9 ± 0.1*, ^†^
Euhydration control	36.8 ± 0.1	37.8 ± 0.1	38.1 ± 0.05*	38.2 ± 0.05*	38.3 ± 0.1*
Mean skin temperature, °C					
Progressive dehydration	35.9 ± 0.1	36.2 ± 0.2	36.1 ± 0.2	36.3 ± 0.2	36.3 ± 0.2
Euhydration control	35.7 ± 0.1	36.3 ± 0.2	36.1 ± 0.2	36.0 ± 0.2	36.1 ± 0.3

Values are means ± *SEM*. For body mass and rectal and mean skin temperatures, *n* = 8 subjects; for red cell and plasma volumes, *n* = 7 subjects.

* *P* < .05 versus 30 min.

^†^ *P* < .05 versus euhydration control.

### 3.2 Systemic and peripheral hemodynamics

**Figure 3:**
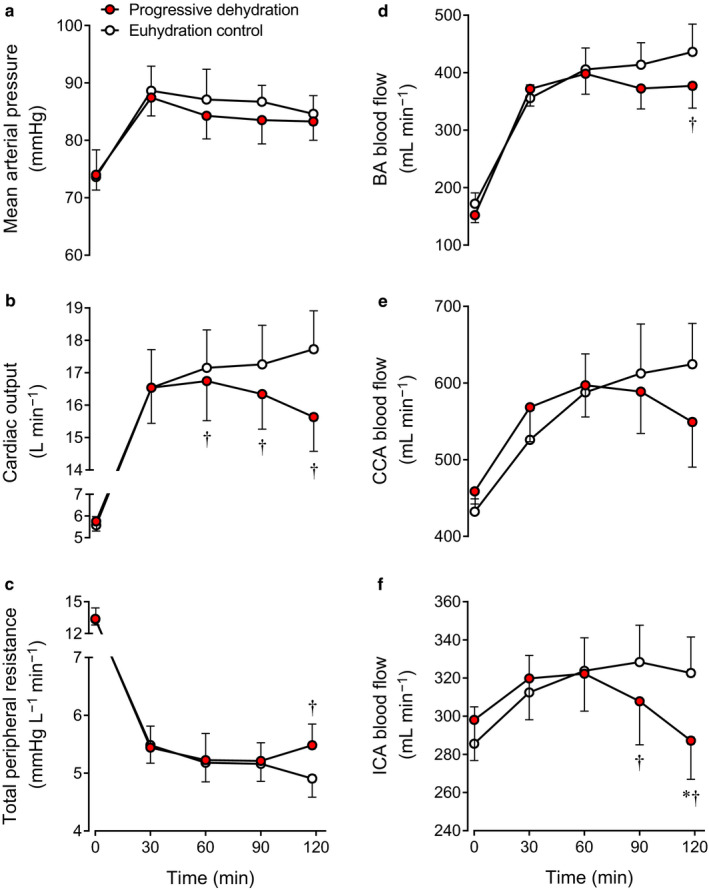
Systemic (a–c) and peripheral (d–f) hemodynamics at rest and during prolonged exercise in the progressive dehydration and euhydration control trials. Data are means ± *SEM* for 8 subjects. *p < .05 versus 30 min; ^†^ *p* < .05 versus euhydration control

$\dot{Q}$ was similar between trials during the early phase of exercise (i.e., 30 min) but was 2.1 ± 0.3 L/min lower at the end of exercise in the dehydration compared with the euhydration trial (15.6 ± 1.1 vs. 17.7 ± 1.2 L/min, *p* < .01; Figure 3b). MAP, however, was similar in both trials (at the end of exercise, *p* = .19; Figure 3a), indicating that TPR was higher at the end of exercise in the dehydration compared with the euhydration trial (5.5 ± 0.4 vs. 4.9 ± 0.3 mmHg/L min^−1^, *p* < .01; Figure 3c), whereas systemic vascular conductance, the inverse of TPR, was reduced (189 ± 14 vs. 210 ± 14 ml/min mmHg^−1^, *p* < .01).

BA blood flow was 14% lower at the end of exercise in the dehydration compared with the euhydration trial (377 ± 39 vs. 436 ± 48 ml/min, *p* < .05; Figure 3d) caused by constriction of the lower BA diameter (0.44 ± 0.01 vs. 0.46 ± 0.02 cm, *p* < .05) while blood velocity was similar (*p* = .32). During the second hour of exercise, CCA blood flow remained higher than at rest in the euhydration trial, whereas in the dehydration trial, CCA blood flow returned gradually to the resting level (Figure 3e). Similar to the changes in BA dynamics, CCA diameter was lower (0.56 ± 0.02 vs. 0.58 ± 0.02 cm, *p* < .05) while blood velocity was maintained (*p* = .33). A similar pattern was observed in ICA blood flow and it was lower at the end of exercise in the dehydration compared with the euhydration trial (287 ± 20 vs. 323 ± 19 ml/min, *p* < .05; Figure 3f) in association with a lower ICA diameter (0.45 ± 0.02 vs. 0.46 ± 0.01 cm, *p* < .05) but an unchanged blood velocity (*p* = .14). BA vascular conductance was also lower at the end of exercise in the dehydration compared with the euhydration trial (4.6 ± 0.5 vs. 5.2 ± 0.6 ml/min mmHg^−1^, *p* < .05). A similar trend was observed at 118 ± 1 min in ICA vascular conductance (3.5 ± 0.3 vs. 3.9 ± 0.3 ml/min mmHg^−1^, *p* = .05) while CCA vascular conductance was maintained (6.7 ± 0.8 vs. 7.4 ± 0.7 ml/min mmHg^−1^, *p* = .15).

### 3.3 LV volumes, heart rate, peripheral beat volume, and intraventricular pressure gradients

**Figure 4:**
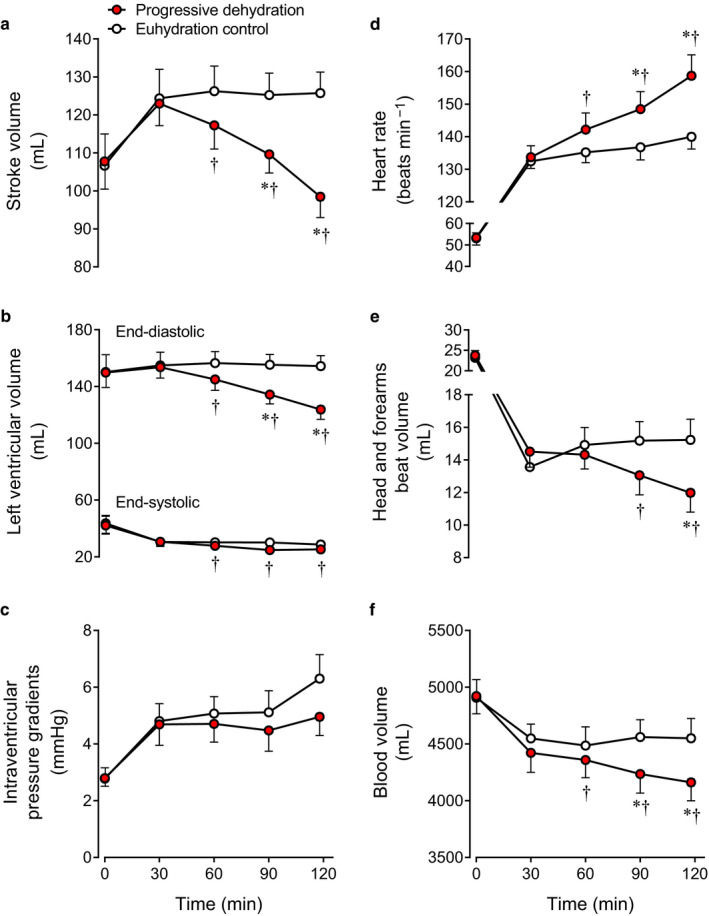
Stroke volume (a), left ventricular (LV) volumes (b), intraventricular pressure gradients (c), heart rate (d), peripheral beat volume (e), and blood volume (f) at rest and during prolonged exercise in the progressive dehydration and euhydration control trials. Cardiac images for intraventricular pressure gradients were successfully analyzed in six of the eight subjects, and blood samples were successfully obtained in seven of the eight subjects. Therefore the intraventricular pressure gradients data and blood volume data are from those six and seven subjects, respectively. The other parameters are from eight subjects. Data are means ± *SEM*. *p < .05 versus 30 min; ^†^ *p* < .05 versus euhydration control

SV decreased progressively during prolonged exercise with dehydration (−27 ± 3 ml vs. euhydration at the end of exercise, *p* < .01; Figure 4a). The lower SV was solely related to reduced LVEDV (−31 ± 4 ml vs. euhydration, *p* < .01; Figure 4b), as LVESV decreased (−3 ± 1 ml vs. euhydration, *p* < .05; Figure 4b). During exercise, HR increased progressively in the dehydration trial, but remained stable in the euhydration trial such that HR at the end of exercise was + 19 ± 4 beats/min higher than the euhydration trial (*p* < .01; Figure 4d). Head and forearms beat volume, an index of peripheral beat volume or venous return, decreased progressively with dehydration during the second hour of exercise and it was −3.3 ± 0.8 ml lower at the end of exercise in the dehydration compared with the euhydration trial (12.0 ± 1.2 vs. 15.2 ± 1.3 ml, *p* < .01; Figure 4e). The intraventricular pressure gradients from the LV base to the apex were similar between the dehydration and euhydration conditions both at rest and during exercise (main effect of hydration *p* = .22; time × hydration interaction effect *p = *.58; Figure 4c).

### 3.4 LV twist mechanics and strains

**Figure 5:**
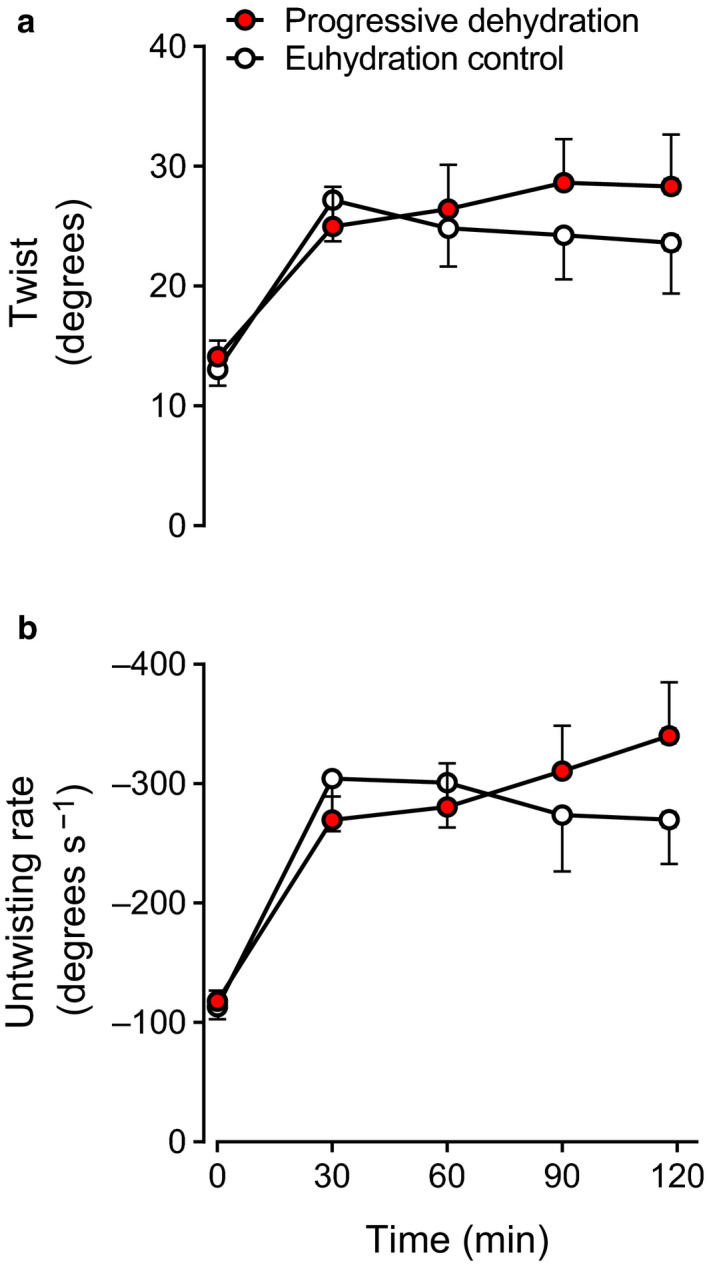
LV twist (a) and untwisting rate (b) at rest and during prolonged exercise in the progressive dehydration and euhydration control trials. Data are means ± *SEM* for eight subjects

LV twist did not change significantly during prolonged exercise in either condition, but tended to be higher at the end of exercise with dehydration (28 ± 4 vs. 24 ± 4 degrees, *p* = .09; Figure 5a). A similar trend was observed at 118 ± 1 min in the LV untwisting rate response (−340 ± 45 vs. −270 ± 37 degrees/s, *p* = .06; Figure 5b). Peak longitudinal, radial, and circumferential strains remained stable throughout exercise in both trials (*p* > .05; Table 2).

**TABLE 2:** Peak systolic and diastolic LV rotation and strain parameters at rest and during prolonged exercise in the heat

	Exercise time (min)				
	Rest	30	60	90	118 ± 1
Systolic component					
Basal rotation, degrees					
Progressive dehydration	−4.2 ± 1.2	−9.9 ± 2.6	−10.1 ± 2.7	−12.7 ± 2.7	−11.8 ± 2.4
Euhydration control	−3.5 ± 1.0	−11.0 ± 2.4	−10.9 ± 2.1	−10.7 ± 2.5	−9.8 ± 2.5
Apical rotation, degrees					
Progressive dehydration	10.2 ± 1.3	17.3 ± 2.9	19.7 ± 2.7	18.6 ± 2.6	19.1 ± 2.9
Euhydration control	10.1 ± 1.5	17.8 ± 2.4	16.0 ± 1.6	16.1 ± 2.1	16.3 ± 2.5
Longitudinal strain, %					
Progressive dehydration	−18.9 ± 0.6^†^	−22.8 ± 0.8	−21.6 ± 0.8	−22.0 ± 0.8	−21.9 ± 0.6
Euhydration control	−18.1 ± 0.7	−22.2 ± 0.6	−23.5 ± 1.0	−22.3 ± 0.6	−22.9 ± 1.0
Basal radial strain, %					
Progressive dehydration	54.7 ± 5.4	39.2 ± 7.2	39.6 ± 8.2	38.0 ± 5.7	38.4 ± 7.0
Euhydration control	50.7 ± 6.0	38.5 ± 8.5	40.4 ± 5.2	39.5 ± 5.5	40.0 ± 6.6
Apical radial strain, %					
Progressive dehydration	22.9 ± 5.3	34.6 ± 4.9	36.4 ± 6.0	42.5 ± 6.6	39.8 ± 6.3
Euhydration control	24.4 ± 5.6	41.5 ± 8.7	38.8 ± 4.6	34.0 ± 4.5	43.9 ± 6.5
Basal circumferential strain, %					
Progressive dehydration	−18.9 ± 1.4	−18.8 ± 2.1	−18.7 ± 2.0	−18.5 ± 1.6	−18.1 ± 1.7
Euhydration control	−18.0 ± 1.7	−19.4 ± 2.0	−19.2 ± 1.8	−19.5 ± 1.5	−20.3 ± 1.6
Apical circumferential strain, %					
Progressive dehydration	−26.2 ± 1.2	−36.3 ± 1.7	−35.4 ± 2.3	−37.1 ± 2.5	−35.3 ± 1.4
Euhydration control	−25.8 ± 1.7	−37.0 ± 2.8	−36.3 ± 2.7	−34.7 ± 2.4	−34.9 ± 1.8
Diastolic component					
Basal rotation rate, degrees s^−1^					
Progressive dehydration	61 ± 4^†^	123 ± 13	130 ± 20	167 ± 24^†^	161 ± 25
Euhydration control	51 ± 4	139 ± 17	137 ± 15	139 ± 18	137 ± 16
Apical rotation rate, degrees s^−1^					
Progressive dehydration	−80 ± 7	−199 ± 21	−220 ± 28	−218 ± 30	−229 ± 31
Euhydration control	−84 ± 11	−227 ± 35	−193 ± 26	−200 ± 32	−183 ± 30

Values are means ± *SEM*; *n* = 8 subjects.

^†^ *p* < .05 versus euhydration control.

### 3.5 Effective arterial elastance and LV end‐systolic elastance

**Figure 6:**
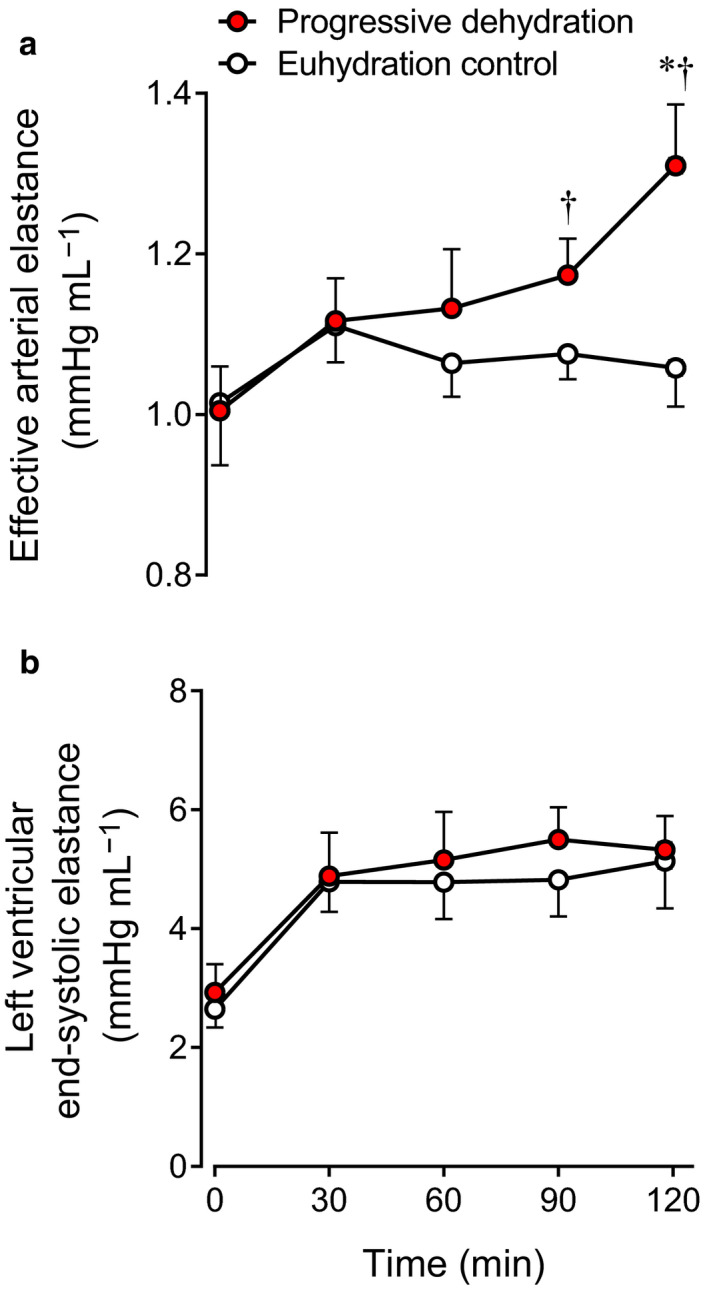
Effective arterial elastance (a) and LV end‐systolic elastance (b) at rest and during prolonged exercise in the progressive dehydration and euhydration control trials. Data are means ± *SEM* for eight subjects. *p < .05 versus 30 min; ^†^ *p* < .05 versus euhydration control

Effective arterial elastance increased progressively with dehydration during the second hour of exercise and it was 19% higher at the end of exercise in the dehydration compared with the euhydration trial (1.31 ± 0.08 vs. 1.06 ± 0.05 mmHg/ml, *p* < .01; Figure 6a). LV end‐systolic elastance remained stable throughout exercise and was similar in both trials (*p* > .05; Figure 6b).

### 3.6 Relationships between SV and LVEDV and between LVEDV and blood volume, peripheral beat volume, or LV filling time during exercise

**Figure 7:**
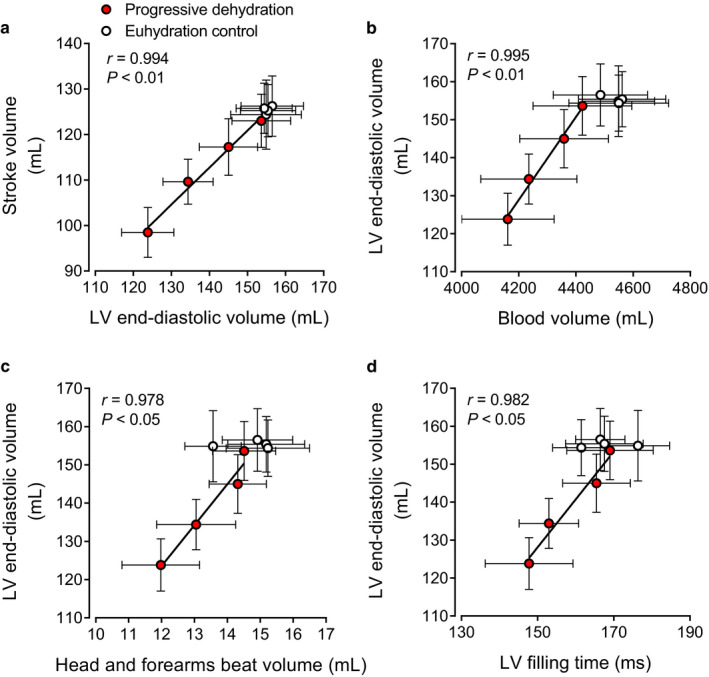
Relationships between stroke volume and LV end‐diastolic volume (a) and between LV end‐diastolic volume and blood volume (b), head and forearms beat volume (c), or LV filling time (d) during prolonged exercise in the progressive dehydration and euhydration control trials. Data are means ± *SEM* for seven (blood volume) or eight (the other parameters) subjects. Lines are regression lines

A significant correlation was observed between reductions in SV and reductions in LVEDV (*r* = .994, *p* < .01; Figure 7a) during prolonged exercise with dehydration. Similarly, the progressive decline in LVEDV correlated strongly with decreases in blood volume (*r* = .995, *p* < .01; Figure 7b), head and forearms beat volume (*r* = .978, *p* < .05; Figure 7c), and LV filling time (*r* = .982, *p* < .05; Figure 7d).

## 4 DISCUSSION

This study carefully manipulated hydration status during intense prolonged whole‐body exercise in the heat to examine its effects on LV volume and mechanics as well as peripheral and systemic hemodynamics and thereby gain insight into the peripheral and central mechanisms underpinning dehydration‐induced cardiovascular strain. The main finding was that the compromised $\dot{Q}$ due to reduced SV with progressive dehydration and hyperthermia compared to euhydration control was solely due to reduced LVEDV, as LVESV decreased, LV twist and untwisting rates tended to increase and left intraventricular pressure gradients were maintained. The lower preload of the heart was in turn associated with reductions in blood volume and peripheral beat volume suggestive of reduced venous return and a tachycardia‐induced shortening of LV filling time. Therefore, the findings suggest that the decline in SV and $\dot{Q}$ with dehydration and hyperthermia during prolonged whole‐body exercise is intimately related to the lowering in LV filling secondary to compromised venous return and diminished cardiac filling time, rather than impaired intrinsic systolic or diastolic LV function.

From a conventional cardiac perspective, SV is thought to be determined by intrinsic cardiac factors such as myocardial contractility and extrinsic factors that alter preload and afterload (Rowell, 1993). Speckle tracking echocardiography affords insight into systolic and diastolic muscle function and thus the intrinsic myocardial function during the cardiac cycle beyond the simple global estimates of cardiac volumes and ejection fraction. A salient finding in the present study is that systolic and diastolic cardiac muscle function was preserved during prolonged intense exercise even in the dehydrated condition. Previous studies have shown that, immediately after long‐duration exercise (e.g., marathon race) when body mass is lowered slightly or moderately (~0.5%–2%), LV diastolic function is blunted (George et al., 2005; Nottin et al., 2012; Oxborough et al., 2010), and that LV systolic and diastolic function is depressed accompanying substantial (~3%–4.5%) body mass loss (Douglas et al., 1987; Niemelä et al., 1984; Nottin et al., 2009; Whyte et al., 2000). In contrast, we found that systolic and diastolic LV muscle function, as represented by systolic LV twist and diastolic LV untwisting rate, was not reduced but tended to increase with dehydration during prolonged exercise, which is possibly attributable to the enhanced sympathetic nerve activity (González‐Alonso et al., 1998, 1999). This suggests that the dehydration‐induced reductions in LVEDV and SV are unrelated to depressed intrinsic systolic or diastolic cardiac function. This idea is in agreement with our paralleled observation of the constant LV end‐systolic elastance and left intraventricular pressure gradients suggestive of maintained LV systolic performance and diastolic suction, respectively.

Afterload and preload are widely known extrinsic cardiac factors affecting LV volumes during the cardiac cycle. From the view of the heart, the LV needs to overcome the load imposed by aortic pressure to eject blood into the systemic circulation during systole and adjust its preload and force and rate of contraction to meet the peripheral needs for oxygen and nutrient supply. However, the observed unaltered MAP suggests that the distinct SV responses between hydration conditions are unrelated to differences in afterload. Although significant reductions in MAP have been observed with dehydration and hyperthermia in previous studies (González‐Alonso et al., 1995, 1997, 1998, 2000; Trangmar et al., 2015), the present result during semi‐recumbent cycling agrees well with a study demonstrating that the dehydration‐induced reduction in MAP during upright exercise was abolished during exercise in the supine position (González‐Alonso et al., 1999). The argument that the afterload remained unchanged from the simple measure of MAP needs to be looked at with caution because we also observed a paralleled increase in effective arterial elastance, which is indicative of an augmented net arterial load exerted on the LV with dehydration and hyperthermia. However, the enhancement of LV emptying (i.e., lowered LVESV) in this study suggests that any rise in afterload to the LV was not a factor explaining the SV decline.

Rather, the strong relationship between reductions in SV and decreases in LVEDV supports a critical role of preload in the reductions in SV (Figure 7a). Similar to findings at rest and during single leg knee‐extensor exercise (Stöhr et al., 2011a), we found that a substantial fall in LVEDV but not an increase in LVESV occur with the development of dehydration during prolonged whole‐body exercise. Thus, from the viewpoint of the heart, the reduced preload and LV filling fully accounted for the SV decline. Whether the right ventricle experiences similar reductions in filling could not be directly assessed in this study; however, the observations that the fall in LVEDV was strongly associated with diminished blood volume and peripheral blood flow and beat volume strongly support this notion (see Figure 7b). These observations raise the question of whether direct evidence in the literature substantiates the idea that alterations in vascular volume and flow influence the preload of the heart. In their classic review, Parker and Case reported that acute reductions in blood volume via phlebotomy in a cardiac patient reduce SV and LV end‐diastolic pressure whereas blood reinfusion normalizes these responses (Parker and Case, 1979). Recent studies in healthy young humans, however, reveal that withdrawal of 20% of blood volume (~1.2 L) does not decrease SV or $\dot{Q}$ at rest and during short duration single leg knee‐extensor exercise (González‐Alonso et al., 2006; Roach et al., 1999). These findings indicate that hypovolemia alone does not affect cardiovascular function in conditions of low cardiovascular load. In comparable conditions to this study, however, plasma volume expansion in dehydrated and hyperthermic individuals has been shown to restore one‐half of the SV decline and attenuate the increase in HR such as that $\dot{Q}$ is maintained during prolonged exercise in the heat (Montain and Coyle, 1992a). It therefore seems that the dehydration‐induced blood volume loss can contribute, but does not fully explain, the observed reductions in SV and $\dot{Q}$ during intense prolonged exercise.

The decline in LVEDV with dehydration and hyperthermia was also strongly related to restrictions in LV filling time (Figure 7d), suggesting that the presently observed ~19 beats/min higher HR at 2 hr of exercise in the dehydration compared to control trial might have also influenced the SV response. Human studies showing that raising HR by right atrial pacing reduces SV and LVEDV (Bada et al., 2012; Munch et al., 2014; Parker et al., 1971; Ross et al., 1965; Stein et al., 1966) and lowering HR by β_1_‐adrenergic blockade increases SV (Fritzsche et al., 1999; Trinity et al., 2010) argue strongly for this second possibility. The intimate relationships between the decline in LVEDV and the reductions in peripheral blood flow and beat volume suggest that peripheral vascular factors might have also contributed the reduced preload of the heart. In this context, levels of dehydration and hyperthermia comparable to those observed in this study (i.e., 3.5% body mass loss and +0.8–1.0°C in core temperature) similarly reduced LVEDV (30–33 ml) and SV (21–22 ml) but not $\dot{Q}$ or leg blood flow at rest and during single leg knee‐extensor exercise in the face of a higher elevation in HR (22–36 beats/min) which can lead to a more pronounced reduction in LV filling time (Pearson et al., 2013; Stöhr et al., 2011a). This contrasts starkly with our observation that the lower $\dot{Q}$ with progressive dehydration and hyperthermia was accompanied by diminished non‐exercising limb and brain blood flow during prolonged whole‐body exercise, in agreement with previous findings (González‐Alonso et al., 1995, 1998; Montain and Coyle, 1992b; Trangmar et al., 2015). A significant attenuation is also seen in exercising limb and skin blood flow under these conditions (González‐Alonso et al., 1998). These findings collectively suggest that the combination of dehydration‐induced hypovolemia and tachycardia‐mediated shortening of LV filling contributes to the gradual fall in LVEDV and SV with dehydration and hyperthermia, but the ultimate fall in $\dot{Q}$ occurs only when peripheral perfusion and concomitant venous return to the heart are also blunted owing to the disproportionally greater reduction in LVEDV and SV compared to the increase in HR.

An important question is whether a cause‐and‐effect relationship exists between the reduction in $\dot{Q}$ and the decrease in peripheral blood flow. The prevailing view is that $\dot{Q}$ determines peripheral blood flow distribution, as in this model of cardiovascular control the heart is the pump that provides the total mechanical energy for blood's propulsion (Furst, 2020; Guyton, 1967; Patterson and Starling, 1914; Rowell, 1993). According to this view, the lower peripheral blood flow and venous return occurring during prolonged exercise with dehydration and hyperthermia might be simply a consequence of the lower $\dot{Q}$. An alternative interpretation is based upon the theory proposed more than half a century ago by Guyton (Guyton, 1967) that $\dot{Q}$ is largely unaffected by the activity of the heart, as discussed above in regard to cardiac pacing (Bada et al., 2012; Munch et al., 2014; Parker et al., 1971; Ross et al., 1965; Stein et al., 1966), and that venous return plays a central role in control of $\dot{Q}$ (Joyce and Wang, 2020). Our findings of regional beat volumes provide insight into whether venous return is compromised when peripheral blood flow is reduced with dehydration and hyperthermia. The estimates including exercising limbs suggest that beat volumes through the head, forearms and exercising legs were reduced by ~20 ml. This value is consistent with the ~22 ml average reduction in femoral beat volume directly observed in our previous study measuring femoral venous flow during prolonged upright cycling (González‐Alonso et al., 1998). Visceral blood flow is also likely to decrease in these conditions (González‐Alonso et al., 1998; Rowell et al., 1965). Thus, it is quite plausible that the restricted peripheral blood flow is associated with reductions in venous return to the heart and these explain a large portion of the observed ~30 ml fall in LVEDV and SV and thus $\dot{Q}$.

**Figure 8:**
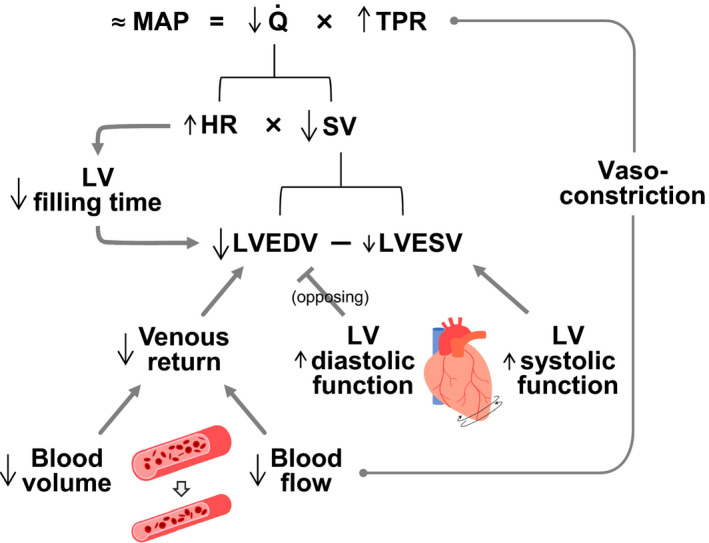
Schematic illustration of the impact of dehydration and hyperthermia on cardiovascular function during whole body exercise according to the hydrodynamic equivalent of Ohm's law. This states that perfusion pressure (MAP assuming right atrial pressure is 0 mmHg) equals flow ($\dot{Q}$) times total peripheral resistance (TPR). Reductions in $\dot{Q}$ due to diminished SV are associated with increases in TPR, as MAP is maintained. The SV decline in turn is solely the results of lower LV end‐diastolic volume (LVEDV), as LV end‐systolic volume (LVESV) and systolic function are not impaired. Lower total blood volume and peripheral vasoconstriction (and/or reduced peripheral perfusion) diminish venous return to the heart and thereby reduce LVEDV. Concomitant tachycardia‐mediated restrictions in LV filling time also contribute to the reduction in LVEDV. Increases in LV diastolic function blunts the effects of hypovolemia, tachycardia and vasoconstriction thereby preventing greater reductions in LVEDV

Granting the possibility that the lower venous return might be simply a consequence of the lower $\dot{Q}$ cannot be ruled out, evidence from studies manipulating peripheral blood flow provides crucial insight into the alternative view that factors controlling the peripheral circulation largely determine the output of the heart (Furst, 2020; Guyton, 1967; Joyce and Wang, 2020). Human investigations using pharmacologically‐induced limb vasoconstriction (via intra‐arterial infusion of adenosine and the sympathomimetic agent tyramine, or the combined blockade of prostaglandins and nitric oxide using N^G^‐monomethyl‐L‐arginine and indomethacin infusion) show decreases in $\dot{Q}$ in proportion to the decrease in limb blood flow (Mortensen et al., 2007; Rosenmeier et al., 2008), whereas limb vasodilation (via intra‐arterial infusion of ATP and other nucleotides) leads to a proportional increase in limb blood flow and $\dot{Q}$ (González‐Alonso et al., 2008; Rosenmeier et al., 2008). Superimposition of intra‐femoral artery ATP infusion during exercise also induces further peripheral vasodilatation and increases in exercising limb hyperemia and $\dot{Q}$ (Calbet et al., 2006; Rosenmeier et al., 2004). However, comparable infusion of ATP into the femoral vein does not change limb blood flow or $\dot{Q}$ (González‐Alonso et al., 2008), suggesting that peripheral vascular mechanisms causing vasodilatation and concomitant increases in limb blood flow and venous return trigger the $\dot{Q}$ response. Although whether pharmacologically‐induced peripheral vasodilatation can restore $\dot{Q}$ during exercise in dehydrated and hyperthermic individuals warrants investigation, there is compelling evidence that factors responsive to dehydration such as vascular fluids, blood gases and temperature can drastically alter exercising limb vascular tone, blood flow and $\dot{Q}$ (Chiesa et al., 2015; González‐Alonso et al., 2006; Roach et al., 1999). In that light, the reductions in forearm and brain blood flow with progressive dehydration in the present study were associated with a decrease in regional vascular conductance underpinned by decreases in vessel diameter. Taken together, these findings support the hypothesis that the peripheral mechanisms restricting blood flow with dehydration and hyperthermia due to augmented vasoconstriction play a role in the impaired central hemodynamics through reducing venous return and LV filling (Figure 8).

### 4.1 Limitations and methodological considerations

In this study, we did not directly measure venous blood flow toward the heart (e.g., flow in the inferior and superior vena cava) or right heart volume and function to exclude alterations in pulmonary hemodynamics as a factor influencing the SV and $\dot{Q}$. To estimate venous return response, we obtained peripheral (head and forearms) beat volume as calculated previously (Elstad et al., 2009; Trangmar and González‐Alonso, 2017) based on an assumption that it tracks changes in the amount of venous blood volume moving each cardiac cycle under steady‐state condition. Our interpretation of the venous return response was based not only on this index but also on the echocardiographic data (i.e., LVEDV) and thus our conclusion about venous return remains intact. Moreover, this notion is consistent our direct measurements of exercising leg blood flow in the femoral vein with thermodilution (González‐Alonso et al., 1998). Nevertheless, it would be important to follow up with specific venous flow measurements to confirm the present observations.

## 5 CONCLUSIONS

The present findings demonstrate that, during prolonged whole‐body exercise in humans, the combination of dehydration and hyperthermia leads to impaired SV and ultimately $\dot{Q}$ because of a reduced LV filling. The decreased preload of the heart is in turn associated with concomitant hypovolemia, reduced venous return possibly due to blunted blood perfusion induced by enhanced peripheral vasoconstriction, and diminished cardiac filling time accompanying tachycardia. In contrast, impaired intrinsic myocardial contractility and relaxation do not appear to be the factor responsible for the cardiovascular strain accompanying progressive dehydration and hyperthermia during prolonged exercise in the heat. These findings highlight the importance of peripheral mechanisms in cardiac performance during intense exercise.

## CONFLICT OF INTEREST

No conflicts of interest, financial or otherwise, are declared by the author(s).

## AUTHOR CONTRIBUTIONS

K.W., E.J.S., and J.G.‐A. conceived and designed the study. K.W., K.A., S.W., and J.G.‐A. were involved in data collection and analysis. All authors interpreted results. K.W., E.J.S., and J.G.‐A. drafted the article and critically revised for important intellectual content. All authors approved the final version of the manuscript.
